# Balancing sweetness: Regulatory insights into sugar accumulation in peach fruit

**DOI:** 10.1093/plphys/kiaf247

**Published:** 2025-06-07

**Authors:** Saadia Bihmidine, Thu M Tran

**Affiliations:** Department of Biology, University of Dallas, Irving, TX 75062, USA; Assistant Features Editor, Plant Physiology, American Society of Plant Biologists; Cold Spring Harbor Laboratory, Cold Spring Harbor, NY 11724, USA

Sugars are essential to plant metabolism, serving as energy sources, metabolic intermediates, and signaling molecules during the development and ripening of fleshy fruits (Aslam et al. 2019). In peach (*Prunus persica*), sucrose is the predominant sugar at maturity, strongly influencing fruit quality and consumer preference (Cirilli et al. 2016). Although significant progress has been made in characterizing sugar metabolism enzymes and transporters, including members of the Major Facilitator Superfamily (MFS), the regulatory networks governing sugar accumulation in fruit crops are still not fully understood (Ren et al. 2023). Understanding these mechanisms is important to improving fruit quality through breeding programs.

Sugar partitioning between source and sink tissues is tightly regulated through diverse transporter families that act across various developmental stages (Aslam et al. 2019). A recent study by Yang et al. (2025), published in *Plant Physiology*, identified MFS transporters with distinct spatial and developmental expression patterns, highlighting their key roles in sucrose accumulation in peach fruit. By combining spatial metabolomics, transcriptomics, and association genetics, they identify 2 key players in sugar partitioning: *PpERDL16-1*, a facilitator of sucrose accumulation, and *PpTST1*, a vacuolar transporter previously associated with fruit sweetness (Ren et al. 2023; Liu et al. 2025). Notably, they revealed that genetic variants upstream of *PpTST1* modulate its expression, leading to variations in fruit sugar content.

Yang et al. identified 67 mFS transporter genes in the peach genome and classified them into 8 subfamilies. They performed functional characterization focusing on key candidates, including Early Response to Dehydration Like (ERDL) transporters, Tonoplast Sugar Transporters (TST), Polyol/Monosaccharide Transporters (PMT), and Sucrose Transporters (SUT). Among these, 2 distinct sugar transporter genes, *PpERDL16-1* and *PpTST1*, showed strong positive correlations with sucrose accumulation, while the other 2 sugar transporters, *PpPMT5-1* and *PpSUT4*, appeared to limit sugar transport. These results were validated through overexpression and silencing experiments in transient systems, confirming their distinct roles in regulating sucrose levels. Overexpression of *PpERDL16-1* transporter enhanced sugar accumulation, whereas *PpERDL6* and *PpPMT5-2* transporter silencing reduced it (Fig. 1A).

**Figure 1.**
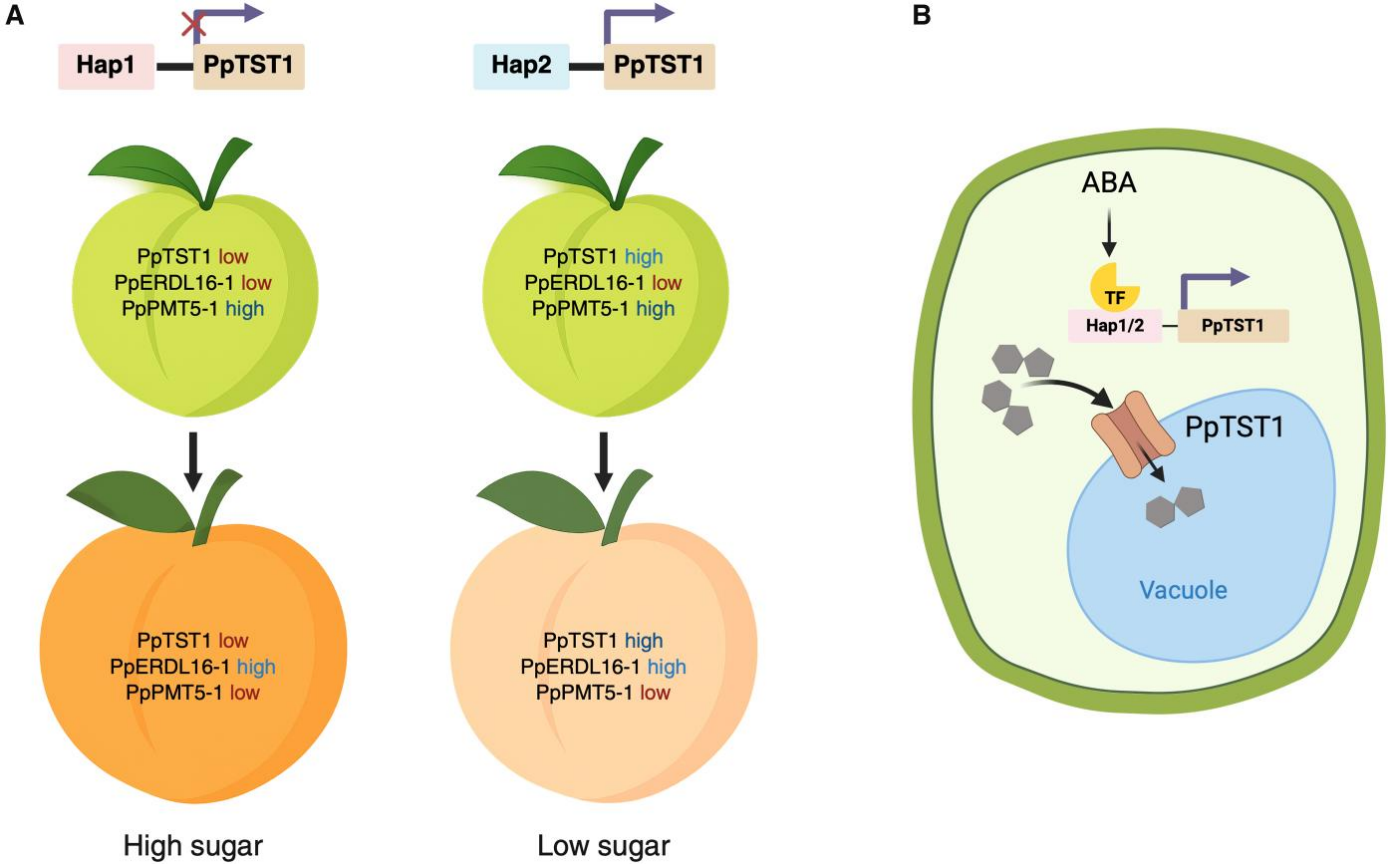
Model illustrating the role of MFS transporters in regulating sucrose accumulation in peach fruit. **A)** Differential expression of sugar transport-related genes in peach fruit under 2 haplotypes, *Hap1* and *Hap2*, at premature (green) and mature (orange) stages. *Hap1* is associated with low expression of PpTST1, leading to high sugar content at maturity. In contrast, *Hap2* promotes PpTST1 expression, leading to reduced sugar accumulation. **B)** Proposed cellular model where ABA signaling and ABA-related transcription factors (TF) influence PpTST1 transcription in a haplotype-dependent manner. PpTST1, a tonoplast-localized MFS transporter, mediates sugar accumulation in the vacuole, contributing to the final fruit sugar content. Figure was created in BioRender (No. FA289D610 V).

To investigate the spatial dynamics of sugar distribution, Yang *et al*. used the matrix-assisted laser desorption/ionization MS imaging metabolomics. This analysis revealed tissue-specific patterns of sucrose accumulation during later stages of fruit development. Notably, *PpTST1* exhibited high expression in inner fruit tissues, supporting its proposed function in vacuolar sugar transport and redistribution (Fig. 1A). These findings highlight the importance of spatiotemporal regulation of sugar transport within fruit tissues. Coordinated regulation of sugar metabolism and transport is essential for sugar accumulation in fruits; however, the underlying mechanisms remain partially understood (Ren et al. 2023). Recent advances in understanding the regulation and manipulation of sugar transporter expression and activity have provided new insights into sucrose loading in source tissues and unloading in sink tissues (Braun 2022).

An interesting aspect of this study was the identification of a noncoding SNP upstream of *PpTST1* that correlates with both gene expression and sugar accumulation. The 2 haplotypes exhibited opposing sugar phenotypes: Hap1 was linked to reduced *PpTST1* expression but higher fruit sugar content, whereas Hap2 was linked to higher expression but lower sugar levels (Fig. 1B). Despite this, silencing *PpTST1* led to reduced sucrose accumulation, suggesting that precise regulation, rather than simple upregulation or downregulation, is necessary for optimal sugar accumulation. Similar balance effects have been observed in other crops, where the interplay between sugar loading and retention fine-tunes sweetness (Zhang et al. 2023). Additionally, although *PpTST1* upregulation increased sugar content, it did not consistently alter the soluble solids content, suggesting the involvement of other regulatory components. These results challenge the assumption that sugar content alone determines sweetness, highlighting the complexity of metabolic balance during fruit development.

The authors further proposed that abscisic acid (ABA) signaling may modulate *PpTST1* activity, as previously observed in apple (*Malus domestica*) and melon (*Cucumis melo*) (Gao et al. 2023; Zhang et al. 2023). However, our understanding of hormone-mediated transcriptional regulation of sugar transporters in fruits remains limited. Given ABA's established roles in fruit ripening and stress responses, it stands as a plausible upstream regulator of sugar partitioning (Fig. 1B). Further investigation into the interaction between hormonal signaling and sugar transporters will help clarify this regulatory network.

Altogether, this study advances our understanding of sugar partitioning in peach fruit, highlighting the combined influence of transporter activity and regulatory sequence variation. The identification of noncoding variants that regulate *PpTST1* expression presents promising avenues for molecular breeding strategies that enhance sweetness without compromising other traits, such as organic acid balance or water content (Luo et al. 2024).

Future research should expand to include transcriptional networks that coordinate sugar metabolism and transport, as well as how environmental signals shape this landscape. It also remains unclear whether these mechanisms are conserved across fruit crops or represent lineage-specific adaptations. As researchers continue to untangle the regulatory architecture of sugar accumulation, the integration of genomics, metabolomics, and hormone biology holds great potential for improving fruit quality through targeted breeding.

## Data Availability

All data discussed in this article can be accessed in the original publication by Yang et al. (2025).
